# Arthroplasty after septic arthritis of the native hip and knee: retrospective analysis of 49 joints

**DOI:** 10.5194/jbji-7-81-2022

**Published:** 2022-04-14

**Authors:** Elodie Portier, Valérie Zeller, Younes Kerroumi, Beate Heym, Simon Marmor, Pascal Chazerain

**Affiliations:** 1 Centre de Référence des Infections Ostéo-Articulaires Complexes (CRIOAC), Groupe Hospitalier Diaconesses Croix Saint-Simon, Paris, France; 2 Service de Rhumatologie, Groupe Hospitalier Diaconesses Croix Saint-Simon, Paris, France; 3 Service de Médecine interne et Infectiologie, Groupe Hospitalier Diaconesses Croix Saint-Simon, Paris, France; 4 Service de Chirurgie Osseuse et Traumatologique, Groupe Hospitalier Diaconesses Croix Saint-Simon, Paris, France; 5 Laboratoire de Biologie Médicale, Groupe Hospitalier Diaconesses Croix Saint-Simon, Paris, France

## Abstract

**Background**: Arthroplasty after septic arthritis (SA) treatment raises diagnostic and therapeutic questions. The main objective was to evaluate infection-free survival of patients undergoing total knee arthroplasty (TKA) or total hip arthroplasty (THA) post-SA. Other objectives were to describe the population's characteristics, surgical strategies, results of preoperative examinations and cultures of intraoperative samples taken at implantation, and postoperative antibiotic therapy. **Methods**: This is a retrospective, observational, monocenter study, from January 2005 to May 2019, including all patients undergoing TKA or THA with prior or ongoing SA in the same joint. Infection–free survival was analyzed and reported. **Results**: Forty-seven patients, 29 men, 49 joints operated on (30 knees, 19 hips), were included. Median SA-to-arthroplasty interval was 32 [1–216] weeks. It was <2 years for 43 joints and <6 months for 19 joints. Six patients underwent arthroplasty while still on SA treatment. One-stage arthroplasty was done for 43 joints and two-stage arthroplasty for 6 joints. Eight (16 %) cultures of intraoperative specimens were positive. Median durations of postoperative antibiotic therapy were 10 d for sterile cultures and 82 d for those that were positive. At 2 years, infection-free survival rate was 95.9 % ( ±0.02 ). After a median follow-up of 47 [18–142] months, no SA relapse was observed, but five patients developed new periprosthetic joint infections (PJIs) with a different microorganism. **Conclusion**: Arthroplasty may be a post-SA option, even within a short period of time. One-stage arthroplasty can be done if synovectomy is thorough, intraoperative samples are taken and antibiotics are administered until those culture results become available. We observed no SA relapse, but new PJIs occurred.

## 1 Introduction

The incidence of septic arthritis (SA) is estimated at 4–10 per 100 000 cases per year in Europe, with an increased risk for patients with diabetes, immunocompromised status, underlying joint disease or prior intra-articular corticosteroid injection(s) (Mathews et al., 2010; Ross, 2017). That incidence is increasing because of population aging, leading to less effective immune-system responses; having more osteoarthritis and comorbidity; and higher rates of joint interventions (Geirsson et al., 2008). Major functional impairments such as pain or difficulty walking can persist after SA, with a frequency estimated at 25 %–50 % (Mathews et al., 2010; Chen et al., 2013; Lauper et al., 2018). This risk of functional sequelae is particularly increased when the infection has caused extensive joint destruction and when severe arthropathy is preexisting (especially osteoarthritis, crystal deposition or inflammatory arthropathy). This functional impact can necessitate prosthetic joint replacement, most often the knee or hip.

The incidence of prosthetic knee or hip infections (PJIs, periprosthetic joint infections) is estimated at 1.5 per 1000 persons annually, for a mean risk of ∼ 1 % (Kurtz et al., 2008; Nair et al., 2017). SA is currently considered a factor associated with PJI, with a relative risk of 6.7 in a large cohort study (Lenguerrand et al., 2018).

Arthroplasty after SA raises several diagnostic and therapeutic questions, concerning surgical timing and modalities and perioperative antibiotics. Various strategies are reported (Sultan et al., 2019; Kim, 1991), but the recent international consensus on orthopedic infections (Aalirezaie et al., 2019) underlines the absence of high-level science-based proofs.

The primary objective of this study was to evaluate infection-free survival of patients undergoing arthroplasty post-SA. Secondary goals were to describe these patients' characteristics, microbiological findings of intraoperative samples and antibiotic regimens.

## 2 Materials and methods

### 2.1 Patients

This retrospective, monocenter, observational study was conducted in a French referral center. Patients provided written consent for use of their personal information for research. All patients hospitalized, between November 2005 and May 2019, for total knee arthroplasty (TKA) or total hip arthroplasty (THA), who had had a prior SA or were being treated for an ongoing SA concerning the joint of interest, were included.

The SA diagnosis was retained when the pathogen was isolated from joint-aspirate or intraoperative samples ( n=41 ). Without microbiological intra-articular documentation, the diagnosis was retained for a clinical picture of acute febrile arthritis associated with an elevated CRP ( >10 mg L ${}^{-1}$ , C-reactive protein), inflammatory joint fluid ( >2000 leukocyte count mm ${}^{-3}$ , >70 % of neutrophils), absence of a differential diagnosis and isolation of a microorganism from one or several hemocultures ( n=5 ). Three episodes with a clinical picture of acute SA were classified as undocumented SA, but SA diagnosis was confirmed retrospectively after isolation of a microorganism on intraoperative samples during arthroplasty. Mycobacterial SAs were excluded.

Patients were identified in the referral center's prospective database. The epidemiological characteristics at the time of arthroplasty and information on prior SA(s) were extracted from each patient's medical file. For two patients with two prostheses, each device was analyzed separately.

Infection control before arthroplasty was assessed clinically (no local inflammatory signs), biologically (normal CRP) and radiologically (no progressive osteolysis). Twenty-eight joints had been aspirated preoperatively. No reason was given in the other patient files to explain why they did not undergo preoperative joint aspiration.

### 2.2 Surgical management and antibiotic therapy

The surgical approach in knees without prior scarring was anteromedial, transquadricipital or anterolateral in fixed genu valgum, combined in some cases of significant stiffness with an anterior tibial tuberosity osteotomy. If there was a previous scar, it was removed in order to limit the number of approaches. Prosthesis surgery consisted of complete synovectomy, obtaining three to five intraoperative samples of synovial membrane, debrided tissue, and/or bone specimen for prolonged culture in enriched media (Zeller et al., 2018); prosthesis implantation with no antibiotic-loaded cement fixation; or uncemented prosthesis. No histology was performed.

During arthroplasty, just after specimens were obtained, empirical intraoperative antibiotics, adapted to the initially identified SA-causative pathogen and cutaneous flora, were started. Most patients were treated with intravenous (IV) cefazolin ( n=30 , 61 %) or amoxicillin ( n=7 , 14 %). The others received either vancomycin or daptomycin ( n=3 ) or other beta-lactam antibiotics ( n=9 ). When intraoperative sample cultures were sterile, antibiotics were stopped 7–14 d later or when a microorganism was isolated, and the regimen was adapted for prolonged use.

### 2.3 Outcome

After prosthesis implantation, follow-up lasted at least 2 years, except for two patients who died of a PJI-unrelated cause at 18 and 23 months after implantation. Outpatient visits were scheduled at 3 and 6 months and then at 1 and 2 years after arthroplasty. Follow-up was mainly clinical and radiological. For patients not seen in consultation, information was obtained by phone interviews.

The following events were assessed: PJI, revision arthroplasty for aseptic loosening, or PJI-related or unrelated death.

PJI was suspected in the presence of persistent joint pain, functional disability, and/or local inflammatory signs or sudden onset of signs of SA on the prosthetic joint. The diagnosis was confirmed by cultures of joint aspirates or intraoperative samples, with at least two isolating the same microorganism, in accordance with international guidelines (Parvizi et al., 2018; Della Valle et al., 2011; Ting and Della Valle, 2017). PJIs were classified either as a relapse of SA on the prosthesis, if the same pathogen as for SA was isolated and if there was no evidence for an acute hematogenous PJI spreading from a distant source of infection, or as a new PJI with a different microorganism.

### 2.4 Statistical analysis

All data were analyzed using R version 4.1.1. Descriptive statistics are presented in the form of the number of occurrences and percentage or as the median with minimum and maximum. The Shapiro–Wilk method was used to test data distribution. For bivariate analyses of continuous variables, Student's t tests were carried out for data with a normal Gaussian distribution. Otherwise, the Mann–Whitney U test was employed. The frequency distribution of categorical variables was compared using the chi-square test or the Fisher exact test, whenever appropriate according to the expected cell frequency.

According to the type of the variable of interest, we used either logistic regression or linear regression to search for the link between independent variables and dependent variable.

Infection-free survival analysis was performed using the Kaplan–Meier method. It was expressed as percentage rate with its standard deviation.

## 3 Results

### 3.1 Patient characteristics

Forty-seven patients with 49 joint-prosthesis implantations after SA were included: 30 knees, 19 hips. Their characteristics are reported in Table 1. Two had bilateral SAs: one for both knees and the other for both hips; each underwent two arthroplasties that were performed separately, 8 and 2.5 months apart, respectively.

### 3.2 SA description

Thirty-four (72 %) SA episodes were not initially managed in our center, where patients subsequently consulted for arthroplasty. There was no statistically significant difference between patients managed in and out of our center, when comparing comorbidities, age, sex, resection, time between AS and arthroplasty, intraoperative specimen positivity during arthroplasty, or rate of PJI.

The contamination routes and the pathogens isolated are reported in Table 1. Hemocultures were positive in 15 episodes: 10 *Staphylococcus aureus*; 2 *Streptococcus pneumoniae*; and 1 each with *Streptococcus dysgalactiae*, *Parvimonas micra*, and *Salmonella enteritidis*. Only one patient had *Staphylococcus aureus *endocarditis.

Three SA-causative pathogens were initially undocumented: *Serratia marcescens* was grown from TKA intraoperative specimens in a patient operated for presumed severe osteoarthritis; *Pseudomonas aeruginosa* was isolated from THA intraoperative samples, and a polymicrobial skin flora (*Staphylococcus schleiferi*, methicillin sensitive (MS) *Staphylococcus epidermidis*, *Cutibacterium acnes, Corynebacterium striatum*) was found after TKA intraoperative samples in two patients having been treated for an acute presumed SA.

**Table 1:** Epidemiological and microbiological characteristics of the 47 patients and 49 SA episodes, according to the affected joint.

Characteristic	Knee	Hip	Total, n (%)
Number of SA episodes	30 (61)	19 (39)	49
Number of patients	29 (62)	18 (38)	47
Men	16 (55)	13 (72)	29 (62)
Risk-enhancing comorbidities			
Cancer <5 years	2 (7)	5 (28)	7 (15)
Diabetes	6 (21)	2 (11)	8 (17)
Others ${}^{a}$	2 (7)	2 (11)	4 (9)
≥1 Cardiovascular risk factor ${}^{b}$	22 (76)	13 (72)	35 (74)
Initial management outside our center	22 (73)	12 (63)	34 (72)
SA contamination route			
Hematogenous	5 (17)	7 (37)	12 (24)
Contiguous	2 (7)	4 (21)	6 (12)
Postoperative	8 (27)	0	8 (16)
Post-intra-articular injection	12 (40)	0	12 (24)
Unknown	3 (10)	8 (42)	11 (22)
SA-causing pathogen			
MS* Staphylococcus aureus*	13 (43)	8 (42)	21 (43)
MS coagulase-negative	4 (13)	2 (11)	6 (12)
MR coagulase-negative	1 (3)	1 (5)	2 (4)
*Streptococcus* ${}^{c}$	7 (23)	7 (37)	14 (29)
*Enterobacterium*	2 (7)	1 (5)	3 (6)
*Pseudomonas aeruginosa*	2 (7)	0	2 (4)
*Acinetobacter*	1 (3)	0	1 (2)
Others ${}^{d}$	4 (13)	2 (11)	6 (12)
Polymicrobial SA	4 (13)	2 (11)	6 (12)
Initially unidentified	2 (7)	1 (5)	3 (6)

MS, methicillin sensitive; MR, methicillin resistant; SA, septic arthritis; ${}^{a}$ Other risk factors: human immunodeficiency virus; chronic hepatitis. ${}^{b}$ Cardiovascular risk factors: hypertension, diabetes, dyslipidemia, ischemic cardiopathy. ${}^{c}$ *Streptococcus*: 5 *Streptococcus agalactiae*, 5 non-hemolytic streptococci (4 *Streptococcus mitis*), 2 each *Streptococcus dysgalactiae* or *Streptococcus pneumoniae*. ${}^{d}$ Other microorganisms: *Candida freyschussii*, *Neisseria gonorrhoeae*, *Prevotella bivia*, *Parvimonas micra*, *Corynebacterium striatum*, *Cutibacterium acnes*.

### 3.3 Medical–surgical SA management

Thirty-three patients' SAs (67 %) were managed surgically: 3 extra-articular drainages (one leg, two psoas abscesses), 17 arthroscopic knee lavages ± synovectomy, 7 synovectomies by arthrotomy, 3 lavages ± synovectomy by unknown method and 3 hip resections at once. Fifteen (30 %) SA joints had undergone multiple interventions (range [2–3]), including three joint resections performed after a failed first surgery.

In total, joint resection was done for five hips and one knee: either after several arthrotomies failed for three or immediately for three hip SAs because of extensive infections occurring on degraded joints or in complex medical settings. A spacer was inserted in four. The pathogens responsible were* Staphylococcus aureus* twice and one each for *Salmonella enteritidis*, *Parvimonas micra*, *Prevotella bivia *or polymicrobial infection.

Antibiotics were given to all for a total duration of 6 [1–24] weeks with an IV duration of 4 [1–12.5] weeks.

The clinical SA outcomes were favorable for all but one episode in a man and woman, who had two knee *Streptococcus agalactiae* SA relapses before prosthesis implantation.

### 3.4 Workup before prosthesis implantation

Characteristics of the population and results of the pre-arthroplasty laboratory workup are detailed in Table 2. C-reactive protein exceeded 5 mg L ${}^{-1}$ for 26 (53 %) episodes.

Cultures of preoperative joint aspiration performed in 28 joints grew *Pseudomonas aeruginosa *or MS *Staphylococcus epidermidis* in two asymptomatic patients with the same pathogens as those identified during SA.

Radiographs showed arthropathy existing before the first SA for 16 (33 %) joints.

**Table 2:** Epidemiological, biological, radiological and microbiological characteristics of 49 arthroplasties after septic arthritis, according to the affected joint.

Characteristic	Knee, n=30	Hip, n=19	Total, n=49
Age at arthroplasty, years	65 [47–82]	61 [29–82]	64 [29–82]
SA-to-arthroplasty interval, weeks	45 [4–216]	29 [1–144]	32 [1–216]
C-reactive protein, mg L ${}^{-1}$	5 [0–69]	13 [0–190]	9 [0–190]
Preoperative radiographic findings			
Calcification suggestive of CPPD	2 (7)	0	2 (4)
Osteoarthrosis prior to SA	11 (37)	3 (16)	14 (29)
Appearance of arthritis ± osteitis	15 (50)	16 (84)	31 (63)
Positive intraoperative samples	7 (23)	1 (5)	8 (16)
Same pathogen as SA	2 (29)	0	2 (4)
Different pathogen than SA	3 (43)	0	3 (6)
Intraoperative diagnosis of SA	2 (29)	1 (100)	3 (6)
Postoperative duration of antibiotics, days	10 [7–97]	10 [7–90]	10 [7–97]

Results are expressed as “median [range]” or “number (%)”. CPPD, calcium pyrophosphate crystal deposition; SA, septic arthritis.

### 3.5 Time to arthroplasty and surgical strategy

**Figure 1:**
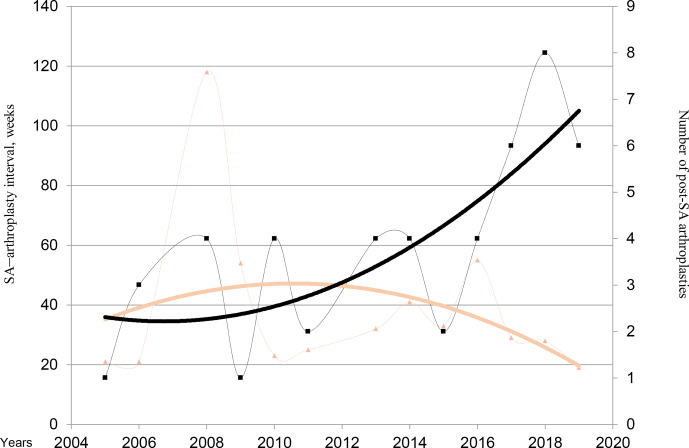
Evolution of the numbers of hip and knee replacements after septic arthritis (SA). Brown triangles and brown line (trend line) represent the median SA–arthroplasty interval. Black square and black line (trend line) represent the number of post-SA arthroplasties per year.

The median interval between the initial SA and prosthesis implantation was 32 [1–216] weeks and decreased over the years, while the number of patients with arthroplasty after SA increased (Fig. 1). However, this association was not statistically significant.

The interval was <2 years for 43 (88 %) joints and <6 months for 19 (39 %). Among the latter, five hips and one knee with major function deterioration underwent arthroplasty while treatment of the infection was still ongoing, after a median of 3.5 [1–6] weeks of antibiotics. The indication for arthroplasty in these cases was based on pain and major functional repercussions despite appropriate antibiotic treatment, mostly because of severe arthropathy secondary to the SA. Two out of six also had advanced pre-existing arthropathy. The pathogens isolated were MS *Staphylococcus aureus *( n=3 ), MS *Staphylococcus epidermidis* ( n=1 ), *Streptococcus mitis* ( n=1 ) and *Streptococcus dysgalactiae* ( n=1 ). Infection control before arthroplasty was assumed in these patients when local signs (swelling, pain, impotence, ± erythema) and CRP had decreased significantly and fever disappeared. None of these patients developed a PJI with a follow-up of 30.5 [25–52] months.

For six other episodes (five knees, one hip), one-stage arthroplasty was done >2 years after SA, with a interval of 32 [24–50] months. Two patients had several SA episodes, with the last being considered cured. Three episodes were suspicious because of preoperative, inflammatory biological syndrome and radiographic images suggestive of osteoarthritis. Only one patient underwent preoperative joint aspiration that yielded sterile cultures. Finally, three of these six patients had positive intraoperative specimens: a patient with clinical, laboratory and radiological signs had persistent *Pseudomonas aeruginosa* SA; *Serratia marcescens* SA was discovered based on the intraoperative samples of a pauci-symptomatic patient; and SA with a different pathogen than the initial episode in a patient without inflammatory syndrome.

Forty-three joints were managed with one-stage arthroplasty. Six further patients had required resection for their SA treatment with spacer implantation in four. Their intervention consisted of removing the spacer, if needed, and prosthesis implantation for all. These patients are considered as having undergone a two-stage procedure.

### 3.6 Intraoperative specimens collected during arthroplasty and postoperative antibiotics

Cultures of intraoperative samples led to the identification of several microorganisms for eight (16 %) joints. Two joint specimens showed persistent infections with the same pathogen: MS *Staphylococcus epidermidis* or *Pseudomonas aeruginosa*. The pathogen differed from that isolated from the initial SA for three joints: *Serratia marcescens*, polymicrobial infection (*Cutibacterium acnes*, *Staphylococcus epidermidis*, MS and methicillin-resistant (MR) *Staphylococcus warneri*, MR *Staphylococcus haemolyticus*), and one fungus (*Cyberlindnera rhodanensis*). Intraoperative samples from the remaining three joints provided a posteriori documentation of the initial SA, with *Serratia marcescens*, *Pseudomonas aeruginosa* or a polymicrobial infection with skin flora.

All patients received empirical antibiotics until the culture results became available, with a median duration of 10 [7–90] d for sterile cultures (with a prolonged antibiotic therapy until 90 d for patients in whom arthroplasty was performed while treatment of the SA was still ongoing). The eight patients with positive cultures were prescribed antibiotics adapted to the pathogens identified for a median duration of 82 [42–92] d.

### 3.7 Outcomes

The follow-up duration post-arthroplasty was 47 [18–142] months. Four patients died of unrelated causes.

Five (10 %) PJI episodes (3 knees, 2 hips) occurred. They are detailed in Table 3. Four had received medical–surgical treatment for their initial SAs, and their specimens obtained during arthroplasty were negative. None had undergone multiple interventions for the initial SA. The last SA was discovered in cultured samples obtained during TKA. All these PJIs were new infections, with three arising more than 2 years post-arthroplasty. Surgical management depended on the acute or chronic nature of the PJI: lavage, debridement, and liner exchange for acute PJI or one-stage complete prosthesis exchange for chronic PJI. Moreover, all patients received prolonged antibiotic therapy (for 6 to 12 weeks). Two patients had multiple PJI episodes that were treated by re-intervention and prolonged suppressive antibiotics.

One patient had PJI involving the same microorganism than the SA (MS *Staphylococcus aureus*), but it was considered a new infection because of a long symptom-free interval between arthroplasty and PJI (5 years) and an acute onset of the infection with portal of entry (foot wound with toe osteoarthritis infection).

**Figure 2:**
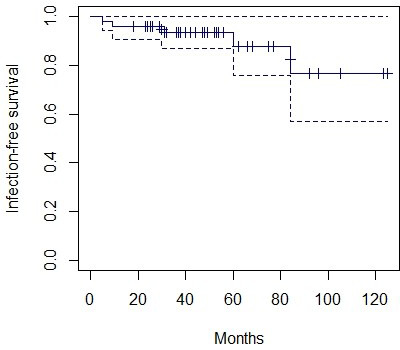
Infection-free survival Kaplan–Meier curve with 95 % confidence interval (dotted lines).

An analysis of infection-free survival after arthroplasty was performed (Fig. 2). The infection-free survival rate at 2 years was 95.9 % ±0.02 .

Only one patient required replacement TKA because of non-septic loosening 10 years after the first arthroplasty.

**Table 3:** Characteristics of post-arthroplasty PJIs following initial SA.

Sex, age (years), site	Comorbidity, SA pathogen	SA–arthroplasty interval (weeks)	Surgery	Months to PJI	PJI pathogen	PJI treatment	Outcome post-PJI
M, 47, knee	None, *Streptococcus agalactiae*	60	1 step	9	MSSA	One-stage exchange arthroplasty; antibiotic therapy for 6 weeks	No recurrence at 25 months
F, 54, knee	Three knee SAs, *S. agalactiae*	118	1 step	5	*Streptococcus oralis*	Lavage and debridement; antibiotic therapy for 6 weeks, then suppressive antibiotics	Three new PJIs with different streptococcal species
F, 62, knee	Severe lymphedema, *Serratia marcescens*	156	1 step	84	MRSE	One-stage exchange arthroplasty; antibiotic therapy for 12 weeks	MRSE relapse, then 2 new hematogenous PJIs with *E. coli*, then MSSA
M, 64, hip	Metastatic anal cancer & diabetes, *Prevotella bivia*	39	2 step	30	*Escherichia* *coli*	One-stage exchange arthroplasty; antibiotic therapy for 6 weeks	No recurrence at 35 months
M, 59, hip	Cirrhosis, MSSA	8	2 step	60	MSSA	Hip resection; antibiotic therapy for 4 weeks	Died of decompensated cirrhosis4 weeks after starting PJI management

F, female; M, male; MSSA, methicillin-sensitive *Staphylococcus aureus*; MRSE, methicillin-resistant *Staphylococcus epidermidis*; PJI, periprosthetic joint infection; SA, septic arthritis.

## 4 Discussion

Prosthesis implantation after SA is becoming more and more frequent, according to national authorities (ANSM, 2015; HAS, 2014). This intervention poses diagnostic and therapeutic questions (Abblitt et al., 2019). We conducted a retrospective study of patients managed in a bone and joint infection referral center, usually with one-stage (88 %) THA or TKA during the first 5 years post-SA.

The first question addresses the risk of PJI caused by either the persistence of the initial SA or a new infection with a different pathogen. This risk is estimated at 1 % in the general population (Kurtz et al., 2008). Lenguerrand et al. (2018) published on 2705 THA infections and concluded that prior SA was a factor associated with PJI with a relative risk of 6.7. The expert panel of the 2019 International Consensus on Orthopedic Infections emphasized the heterogeneity of SA–arthroplasty intervals, joint or bone involvements, the sites, and microorganisms in the publications available, which limited their conclusions and left them open to debate (Aalirezaie et al., 2019). Moreover, a recent case-control study involving 215 primary TKA (Bettencourt et al., 2021) revealed a 6.1-fold increased risk of PJI in patients undergoing TKA with a history of native knee SA when compared with controls undergoing TKA for osteoarthritis, with a cumulative incidence of 9 % at 10 years. Bettencourt et al. (2022) did the same case-control study on the hip and found a 10-fold increased risk of PJI in patients with a history of SA. This risk was more important when arthroplasty was performed within 5 years, highlighted by a 3-fold increase of this risk compared with those in whom arthroplasty was undertaken more than 5 years after SA.

Our results showed that 10 % of our patients having an SA prior to arthroplasty developed PJI, which were always new infections, i.e., with a pathogen or different strain than that causing the SA. The arthroplasty–PJI interval ranged from 5 months to 7 years. For three of the five patients, this new infection was late, appearing >2 years post-arthroplasty, suggesting a hematogenous infection, with local or general susceptibility to infection. Tan et al. (2021) found very close PJI rates in a recent retrospective study (Tan et al., 2021). They observed 25 PJIs (12 %) out of 207 THA or TKA performed after prior SA. Among these were also relapses of the previous infection, as half of their PJI microorganisms were the same as those found during SA and one-third developed PJI within 90 d after arthroplasty. Jerry et al. (1988) suggested a link between local SA severity and the risk of subsequent PJI, with a 4 % reinfection rate of patients with prior SA vs. 15 % with SA and bone involvement. The literature review by the consensus conference experts (Aalirezaie et al., 2019) reported the following PJI rates: 8.26 % for TKAs, 5.2 % for THAs and 6 % globally.

Therapeutic strategies preceding arthroplasty post-SA are poorly defined. Sultan et al. (2019) recommended waiting 2 years to confirm SA cure. The absence of reliable data on the subject was underscored by the 2019 international consensus, but 87 % of orthopedists approved prosthesis implantation post-SA with a minimal interval of 3 months (Aalirezaie et al., 2019). Tan et al. (2021) question specifically that point in their study on 207 arthroplasties performed after prior SA. They found that the optimal threshold for timing of arthroplasty from the initial treatment was 5.9 months, but no difference in the PJI rate was observed when the cohort was dichotomized by this threshold. They concluded that delaying arthroplasty did not appear to reduce the PJI risk. In our study, the delay between SA and arthroplasty decreased over the years (even if it was not statistically significant), while the number of patients with arthroplasty after SA increased. We have not enhanced the ability to detect infection resolution, but our extensive experience in joint arthroplasty and in PJI treatment as well as the general trend among orthopedic surgeons to allow earlier arthroplasty in these cases (Aalirezaie et al., 2019) has certainly contributed to this evolution. Thirty-nine percent of the arthroplasties were performed <6 months after SA. For those 19 patients, 6 underwent arthroplasty while they were still taking antibiotics for the initial SA management because of persistent severe functional deterioration and pain due to extensive joint destruction. Samples obtained during arthroplasty for these six patients were sterile, and none of them developed a PJI with a median follow-up of 30.5 months.

Preoperative workup for our cohort patients included blood counts, CRP, standard X-rays and, most often, joint aspiration to search for persistent infection. Joint aspiration enabled detection of persistent infections in two asymptomatic patients. Although the yield of this examination is not high, we recommend it systematically for patients in this situation. However, the absence of joint fluid can limit its feasibility. In their study, Tan et al. (2021) could not assess the role of joint aspiration as it was performed in only 16.9 % of their patients, but they concluded that CRP or erythrocyte sedimentation rate at the time of arthroplasty had little value in predicting the development of PJI.

During arthroplasty, multiple specimens were obtained without prophylactic preoperative antibiotics to assure optimal conditions for culture. These samples were positive for eight (16 %) joints. Two initial SAs persisted despite the patients being asymptomatic; neither developed infection recurrence of the prosthesis. For the other cultures, three each grew pathogens differing from those isolated from the SA or led to an a posteriori diagnosis of SA. In the study by Ohlmeier et al. (2020), nine (13 %) intraoperative samples grew microorganisms, with subsequent similar antibiotic regimens and no PJIs. The study by Mainard et al. (2021) analyzed the benefit of systematic intraoperative sampling during lower-limb arthroplasties after osteoarticular infection in a retrospective study including 92 patients. They found nearly the same rate of positive cultures (17 %), half with bacteria being the same as the prior infection. They underline that the time from the initial bone and joint infection to the arthroplasty was not associated with positive results. That non-negligible rate of positive samples and the good outcomes from Ohlmeier et al. (2020) and from our patients after adapted treatment of the identified isolated pathogen underline the importance of systematically obtaining intraoperative specimens. Those samples demonstrate persistent infection, even in patients who clinically appeared to be cured with reassuring preoperative laboratory workups.

During surgery, the prosthesis was implanted after synovectomy and sample procurement, usually in one stage and more rarely in two stages. Because of the risk of relapse, some teams systematically perform two-stage arthroplasty for patients with prior SA. At the international consensus conference, 85 % of orthopedists opted for two-stage arthroplasty after active SA and one-stage replacement for quiescent infections (Aalirezaie et al., 2019; Ohlmeier et al., 2020; Bauer et al., 2010). Unfortunately, the distinction between active and quiescent infections was not clear and can be difficult to define in practice. Tan et al. (2021) observed no difference in PJI rate between the one- and two-stage arthroplasty groups. Herein, one-stage arthroplasty seems to have been an effective therapeutic option, including for patients with an ongoing infection. The choice of initial resection arthroplasty was guided in three patients by the initial extent of the infection and osteoarticular destruction or performed in three further patients after failure of several arthrotomies. These patients are considered having undergone a two-stage procedure. A conservative strategy to treat SA was not possible because of a very extensive infection occurring on an already badly damaged joint.

Our study has several limitations. First, its retrospective, monocenter design, with inclusion of a limited number of heterogeneous episodes is limiting, as for most similar investigations (Sultan et al., 2019; Ohlmeier et al., 2020; Bauer et al., 2010; Jerry et al., 1988; Seo et al., 2014; Lee et al., 2002). However, our SA–arthroplasty interval was <5 years and usually <2 years, and we most often treated with one-stage arthroplasty with systematic intraoperative sample collection. Median follow-up was 47 [18–142] months. To avoid recruitment bias and to get a real-life analysis, we did not restrict patient inclusion to those with initial SA who underwent one-stage arthroplasty within 2 years post-diagnosis. Thus, our series included six patients with an SA–arthroplasty interval of >2 years. Their intraoperative sample cultures were positive for half; only one was symptomatic. These observations underscore that the 2-year interval is insufficient to affirm that the SA was cured. The patient's underlying conditions, local signs, radiographic findings, CRP level and joint aspiration are important input items to take into consideration before arthroplasty. Moreover, surgeons must maintain high suspicion whenever performing arthroplasty after SA, and multiple intraoperative samples should be systematically taken in these cases.

Finally, postoperative functional evaluation was beyond the scope of this study.

In conclusion, TKA and THA can be done post-SA, including within the short interval of 3 months or even in some instances during treatment if joint function is severely affected, after collegial discussion and with specific medical–surgical management to prevent recurrences. One-stage arthroplasty is possible, with synovectomy and systematic collection of intraoperative samples, and it must be followed with antibiotics until culture results become available. Our own practices evolved in this way, and we hope that they will also help other teams to manage these situations in a new and different way. These data have to be confirmed subsequently by the constitution of a prospective cohort. Following arthroplasty, prolonged monitoring should be planned, especially when the PJI risk (because of favorable local or general conditions) is elevated.

## Data Availability

The data sets generated and/or analyzed during the current study are not publicly available due to ethical and regulatory restrictions.

## Code availability

Programming codes used for statistical analysis during the current study are available from the corresponding author upon reasonable request.
